# Next-generation sequencing (NGS) for assessment of microbial water quality: current progress, challenges, and future opportunities

**DOI:** 10.3389/fmicb.2015.01027

**Published:** 2015-09-25

**Authors:** BoonFei Tan, Charmaine Ng, Jean Pierre Nshimyimana, Lay Leng Loh, Karina Y.-H. Gin, Janelle R. Thompson

**Affiliations:** ^1^Center for Environmental Sensing and Modelling, Singapore-MIT Alliance for Research and Technology CentreSingapore, Singapore; ^2^Department of Civil and Environmental Engineering, National University of SingaporeSingapore, Singapore; ^3^Singapore Centre on Environmental Life Sciences Engineering, Nanyang Technological UniversitySingapore, Singapore; ^4^School of Civil and Environmental Engineering, Nanyang Technological UniversitySingapore, Singapore; ^5^Department of Civil and Environmental Engineering, Massachusetts Institute of Technology, CambridgeMA, USA

**Keywords:** next-generation sequencing, water quality, fecal indicator, antibiotic resistance, harmful algal bloom, sewage, biodegradation

## Abstract

Water quality is an emergent property of a complex system comprised of interacting microbial populations and introduced microbial and chemical contaminants. Studies leveraging next-generation sequencing (NGS) technologies are providing new insights into the ecology of microbially mediated processes that influence fresh water quality such as algal blooms, contaminant biodegradation, and pathogen dissemination. In addition, sequencing methods targeting small subunit (SSU) rRNA hypervariable regions have allowed identification of signature microbial species that serve as bioindicators for sewage contamination in these environments. Beyond amplicon sequencing, metagenomic and metatranscriptomic analyses of microbial communities in fresh water environments reveal the genetic capabilities and interplay of waterborne microorganisms, shedding light on the mechanisms for production and biodegradation of toxins and other contaminants. This review discusses the challenges and benefits of applying NGS-based methods to water quality research and assessment. We will consider the suitability and biases inherent in the application of NGS as a screening tool for assessment of biological risks and discuss the potential and limitations for direct quantitative interpretation of NGS data. Secondly, we will examine case studies from recent literature where NGS based methods have been applied to topics in water quality assessment, including development of bioindicators for sewage pollution and microbial source tracking, characterizing the distribution of toxin and antibiotic resistance genes in water samples, and investigating mechanisms of biodegradation of harmful pollutants that threaten water quality. Finally, we provide a short review of emerging NGS platforms and their potential applications to the next generation of water quality assessment tools.

## The Role and Precedent of “Proxies” in Water Quality Analysis

Surface freshwaters including lakes, rivers and streams, are important aquatic ecosystems and are a source of drinking water in many countries. In recent decades, increase in human population size and urbanization have exerted immense pressure on the use of these water resources for human recreation and consumption. Contamination by sewage, toxic chemicals, nutrients, and resultant harmful algal blooms can render water unfit for human consumption or recreational activities. Bio-monitoring using sentinel or indicator species in the form of microorganisms and aquatic macroinvertebrates are frequently used by water management authorities to infer water quality, ecosystem health status, and to protect public health from waterborne risks (Carew et al., 2013). The occurrence and abundance of indicator organisms serve as proxies, i.e., easily measured quantities that are correlated to often unknown agents that directly mediate waterborne risk such as pathogens, biotoxins and chemicals. Advances in molecular methods and next-generation sequencing (NGS) have ushered in new opportunities for water quality assessment through analysis of waterborne microbial communities for the development of indicators and sentinels, new markers for microbial source tracking, and observation of microbially mediated processes. However, as these new tools are developed, biases and uncertainties associated with nucleic-acid based methods and NGS must be considered.

For decades, water quality for drinking and recreational purposes has been largely assessed based on culture-based enumeration and detection of fecal indicator bacteria (FIB), e.g., total coliforms, *Escherichia coli*, or *Enterococci*: a practice that has been regarded as the “gold standard” in the assessment of microbial safety of water (Figueras and Borrego, 2010). FIB are at high concentration in human feces, thus their presence in freshwater serves as a proxy for associated pathogens, where risks of exposure and has been shown to correlate to incidence of disease in exposed populations (e.g., Haile et al., 1999; Zmirou et al., 2003; Colford et al., 2007; Harwood et al., 2014). However, these proxies are imperfect as they may be derived from non-human sources and/or may be subjected to ecological or environmental interactions that compromise their predictive power as proxies for pathogens. In addition to predicting contamination from sewage, microbial proxies are used to predict the origin or source of sewage since fecal material of human-origin poses the most significant risk to water quality due to its potential to transmit human pathogens. Research into “source tracking” has established at least two species within the genus *Bacteroides* as indicators of human-origin, which have been shown to associate with and perhaps be symbionts of the human gut (Bernhard and Field, 2000; Shanks et al., 2007, 2009, 2010; Yampara-Iquise et al., 2008). Finally, direct detection and enumeration of specific pathogens such as waterborne *Leptospira, Campylobacter, Legionella*, viruses (e.g., Norovirus) and parasites (e.g., *Cryptosporidium*) enables quantitative microbial risk assessment (QMRA; Hass et al., 2014), where environmental concentrations of pathogens are compared to models of infectivity to characterize the risk to exposed human populations.

Development and validation of methods for enumeration of indicators and/or pathogens presents major challenges. Initial methods for identification of waterborne indicators and pathogens relied on selective culturing and enumeration of presumptive isolates, followed by species confirmation using biochemical, serological or molecular genetic methods. Such efforts lead to the standardization of simple and routine procedures for quantification of FIB, which are relatively easy to culture (Dufour, 1984; Simpson et al., 2002). However, cultivation methods for detection of specific pathogens are often time-consuming, and may fail to detect some organisms due to fastidious growth or requirement for a host (in the case of intracellular pathogens; Evangelista and Coburn, 2010). In addition, some pathogens are known to exist in a dormant, but still infective state that cannot be quantified by standard cultivation-based methods, i.e., the viable but not-culturable (VBNC) state (Ramamurthy et al., 2014).

To circumvent problems associated with quantification of VBNC and otherwise difficult to culture bacteria, cultivation-independent molecular methods were developed for detection and quantification of specific bacteria (recently reviewed by Ramamurthy et al., 2014). Such methods target DNA extracted from environmental and clinical samples which is subsequently subjected to analysis for the presence or abundance of genes from indicator species or pathogens of interest. The small subunit ribosomal RNA gene (SSU rRNA), consisting of the 16S rRNA gene for bacteria and the 18S rRNA gene for eukarya, has emerged as one of the most frequently used target genes for molecular analysis. This is due to its ubiquity in all organisms and sequence structure which includes both highly conserved and variable/hypervariable regions to promote alignment of the DNA sequence across diverse organisms, and allow for more fine-scale taxonomic identification (e.g., Guo et al., 2013). Use of the polymerase chain reaction (PCR) and quantitative PCR (qPCR) targeting regions of the SSU rRNA and functional genes for microbial source tracking and direct detection and quantification of target indicator strains has been recently reviewed by Harwood et al. (2014).

Cultivation-independent analyses based on direct detection of nucleic acids or amplification of targeted genes using PCR circumvent some problems associated with culture-based methods, while raising additional challenges. First, detection and quantification of environmental DNA from individual species is often assumed to be derived from living organisms, however, naked or free DNA may also be detected in such methods, making the correlation between DNA copies and cell abundance imperfect. Second, DNA-based methods used for quantification of microbial risk agents may be confounded by the highly dynamic and diversified genomes of pathogens and prevalence of strain-specific virulence factors. Thus, quantification of pathogenic taxa based on occurrence of biomarker DNA such as the SSU rRNA may not correlate to public health risk if the strain detected lacks virulence genes. Nevertheless, PCR-based detection and quantification of organisms in environmental DNA has proven useful for enumeration of sewage indicators and one DNA-based method is approved for quantification of the fecal indicator *Enterococcus* by the US Environmental Protection Agency (USEPA, 2009, 2013) while additional DNA-based methods are currently under evaluation (Green et al., 2014). A third challenge is raised by the ability of nucleic-acid based analyses to detect as little as one DNA target molecule; thus, it is possible to detect trace levels of nucleic acids from microbial risk agents (e.g., pathogens or virulence factors). Risk assessment frameworks to define thresholds of “acceptable risk” will be necessary before incorporating quantification of microbial risk agents by DNA-based approaches into decision-making for water quality management.

## Brief Review of NGS Technologies and Analysis Methods

Advances in NGS enabling massively parallel analysis of DNA sequence information from PCR amplicons, or environmental nucleic acids, ushers in a new era of proxy development for water quality assessment. In clinical research, massively parallel sequencing (MPS) has been demonstrated as a screening tool used to complement or circumvent conventional diagnostic methods (e.g., culturing, microscopy and Gram-staining) for the detection and identification of etiological agents in disease (Kumar et al., 2013; Salipante et al., 2013). While quantitative tests for water quality assessment (e.g., qPCR, culture-based FIB quantification kits) are appropriate for estimating exposure to biological risk agents or sewage contamination, application of NGS surveys can be a first step to focus on more specific exposure assessment of appropriate targets.

Surveys of waterborne microbial communities using NGS thus far, have relied upon targeted sequencing of the hypervariable regions of SSU rRNA gene (e.g., V1, V3, V4, V6 regions) and Large Subunit (LSU) rRNA gene (e.g., Guo et al., 2013). Apart from SSU and LSU rRNA genes, other genes with taxonomic signals such as *nirS* (denitrification) and *nifH* (nitrogen fixation) indicative of biochemical cycles (e.g., Farnelid et al., 2011; Bowen et al., 2013), as well as plastid SSU rRNA (Steven et al., 2012) have also been adopted for NGS-based microbial profiling.

In early studies of the relationship between waterborne microbial communities and water quality, 454 pyrosequencing (Roche) emerged as the preferred platform of choice (e.g., McLellan et al., 2010; Vandewalle et al., 2012) due to relatively long sequence read lengths (i.e., initial read lengths of 110 bp, now currently ∼1000 bp van Dijk et al., 2014a) and generally better optimized sequencing conditions and bioinformatic workflows (e.g., Sergeant et al., 2012). The Illumina (Solexa) platform was introduced to the market with read lengths of 35 bp with a focus on genome sequencing (van Dijk et al., 2014a). However, as the Illumina technology improved and read lengths achieved by merging paired-end reads began to rival pyrosequencing, it has gained in stature for use as a platform for analysis of environmental samples. Other NGS platforms including Ion Torrent and single molecule real-time sequencing (SMRT) such as the Pacific Biosciences have also been used in amplicon sequencing for microbial community profiling (e.g., Marshall et al., 2012; Yergeau et al., 2012), although these technologies have not been widely adopted. An emerging number of studies in recent years have used the Illumina MiSeq platform in shotgun amplicon sequencing of SSU rRNA (e.g., Newton et al., 2015), with several studies demonstrating improved performances (e.g., sequencing depth, coverage, detection sensitivity, false positive detection) compared to 454 pyrosequencing and Ion Torrent (Loman et al., 2012; Li et al., 2014; Sinclair et al., 2015).

Several open-source bioinformatic platforms including MOTHUR (Kozich et al., 2013) and QIIME (Caporaso et al., 2010), which are among some of the most commonly used software packages in the analyses of amplicon sequences, have been updated to improve on sequence analyses using paired-end sequence data generated from the Illumina platform. As Illumina MiSeq technology has become one of the most widely used sequencing platforms worldwide, and with the announcement by Roche to withdraw the GS FLX 454 pyrosequencing platform, several studies have embarked on further refining data analyses for Illumina platforms by improving on methods in library preparation (Kozich et al., 2013; Esling et al., 2015; Shishkin et al., 2015) and quality control of sequence reads (Kozich et al., 2013; Nelson et al., 2014; Schirmer et al., 2015). The success of using MPS of the SSU rRNA gene for routine monitoring and general sample comparative purposes, however, will hinge on efforts to streamline processes in samples processing, which at the current stage, can vary from one laboratory to another.

Small subunit rRNA gene amplicon sequences generated through MPS are generally clustered into operational taxonomic units (i.e., OTUs) based on nucleotide identity thresholds (e.g., 95–99%). In some cases, however, OTU clustering may fail to segregate highly identical sequence variants into ecologically- or environmentally- relevant groups. A method termed “oligotyping” has been developed to classify and group sequences based on sequence minimum entropy decomposition (MED), at the resolution of single nucleotide polymorphisms (SNPs), and has been shown to be particularly useful in tracing different microbial populations in sewage treatment facilities to their environmental origins (Eren et al., 2013, 2014). Community composition cataloged using NGS can serve as a baseline or reference for monitoring environmental perturbations or biodegradation. Furthermore, NGS-enabled biostatiscal analyses (e.g., principle coordinate analyses, non-metric multidimensional scaling or correspondence analysis) have been adopted to correlate the occurrence and distribution of microbial taxa, OTUs or oligotypes to environmental metadata, thereby allowing identification of oligotypes or OTUs that can serve as bioindicators for environmental quality (e.g.,Yergeau et al., 2012).

In order for NGS to become a useful tool for water quality monitoring purposes, long term sequence data collection and management will be crucial in establishing databases that are important for inter-laboratory data comparison and comparative metagenomics studies. Using a combination of NGS approaches (e.g., metagenomics, metatranscriptomics, single-cell genomics, and comparative genomics) in systematic studies of freshwater microbiomes can be expected to yield a wealth of information crucial in water quality assessment and management.

## Can NGS be Quantitative?

### Massively Parallel Sequencing of SSU rRNA Gene Amplicons

Similar to proxies based on cultivation or cultivation-independent enumeration of indicators and pathogens, MPS of targeted genes such as the SSU rRNA has challenges which will prevent proxies developed with this technology from being 100% accurate. Moreover, the role of NGS in water quality assessment is in its infancy and has not yet been integrated into an epidemiological framework to link trends observed with NGS studies to adverse effects in human populations. One requirement of microbial risk assessment is the ability to accurately quantify biological risks/agents within the framework of QMRA (Hass et al., 2014). While a number of molecular tests including qPCR and digital PCR are known to be highly quantitative, providing results in the form of absolute gene copy number, profiles of species composition generated from amplicon sequencing are generally regarded as being qualitative. Biases in PCR amplification due to secondary structure or GC content of the resulting amplicons, generation of false diversity from sequencing error or chimera formation, choice of primers targeting different SSU rRNA hypervariable regions (Kozarewa et al., 2009; Quail et al., 2012; Kozich et al., 2013; Nelson et al., 2014; van Dijk et al., 2014b; Schirmer et al., 2015), as well as the presence of multiple copies of the SSU rRNA gene in some bacterial species (Angly et al., 2014) all influence the relative abundance of taxa observed by PCR-based methods with more pronounced biases associated with increased PCR cycle numbers (Murray et al., 2015; Schirmer et al., 2015; Sinclair et al., 2015). Sequencing errors associated with reads generated from the Illumina MiSeq platform may also result from differences in library preparation methods (Schirmer et al., 2015). Downstream bioinformatic data processing including methods in chimera removal (Kennedy et al., 2014) and OTU clustering (e.g., UCLAST, CD-HIT, UPARSE, Nelson et al., 2014; Schmidt et al., 2014; Sinclair et al., 2015) can similarly contribute to biases in determining the relative abundance and diversity of microbial taxa. Studies of mock bacterial communities suggest that these variations can sometimes result in inflated OTU numbers and therefore skew estimates of species richness and evenness (Kennedy et al., 2014; Nelson et al., 2014).

Despite the concerns discussed above, several studies have demonstrated that MPS of the SSU rRNA gene does offer a good approximation of the microbial species composition and relative abundance in samples (Ong et al., 2013; Wang et al., 2013), though the accuracy is likely sample-dependent and may be influenced by factors such as methods in library preparation and primer choice, as discussed above. Due to inherent differences in sample types, and associated microbiota, it is likely that no single universal approach can be best applied to all samples types to achieve quantitative measurement (Schmidt et al., 2014; Murray et al., 2015).

### Quantification of Gene Copy Number in Multi-Omics Datasets

Due to concern with biases in PCR amplification, several studies have carried out amplification-independent analysis of microbial communities using SSU rRNA genes extracted from metagenomic datasets. For example, Logares et al. (2014) recently assessed the diversity of marine plankton communities using three different NGS platforms (i.e., metagenomic shotgun sequencing by Illumina HiSeq and Roche 454, and amplicon sequencing of SSU rRNA gene by Roche 454). The diversity and composition of SSU rRNA genes observed in metagenomes prepared by the Illumina HiSeq platform provided higher and more even estimates of community diversity relative to the metagenomes or amplicons sequenced using the Roche 454 platform. In the same study, the relative abundance of microbial taxa observed by Illumina HiSeq was comparable to relative abundances observed by catalyzed reporter deposition fluorescence *in* situ hybridization (CARD-FISH) and flow cytometry (i.e., positive Pearson correlation and *p* < 0.01).

The choice of NGS platform may yield results with varying resolution and quantitative power due to differential sequence throughput and coverage. Frey et al. (2014) recently compared three sequencing platforms (i.e., Roche 454, Illumina MiSeq, and Ion Torrent) to characterize the relative abundance of viral and bacterial pathogens spiked into blood at known, serially diluted, concentrations (i.e., Dengue virus Types 1 and 2; Swine Influenza A, and *Bacillus anthracis*). The blood samples were lysed using commercial kits, and one portion of DNA sample was sequenced using the three independent NGS platforms, while the second portion was subjected to qPCR for the quantification of specific virus and bacterial targets. Sequence reads from NGS were mapped to the reference genomes of the tested organisms, and all three NGS platforms were found to produce results comparable to the qPCR assay for relative quantification. Illumina and Ion Torrent provided the highest sensitivity to detect sequences at the lowest dilutions due to their higher throughput than Roche 454 (Frey et al., 2014).

Several protocols have been developed to quantify absolute gene copy numbers or transcripts within a metagenome or metatranscriptome, respectively (Gifford et al., 2011; Satinsky et al., 2014a,b) by benchmarking to standards that were added to sample prior to total RNA/DNA extraction, library construction, and high throughput sequencing (Gifford et al., 2011). In several metatranscriptomics studies (Gifford et al., 2011; Satinsky et al., 2014a), *in vitro* RNA internal standards were prepared using commercially acquired plasmid DNA, linearized and restriction-enzyme digested, after which plasmid fragments were transcribed *in vitro*. Following this, the absolute copy number of the internal standard was quantified by spectrophotometry before addition into a sample prior to cell lysis and RNA extraction. Benchmarking of sequenced transcripts to the copy number of internal standard was used to estimate sequencing depth and absolute quantity of differential expressed gene or transcript-type. Similarly, DNA standards in known concentration (Satinsky et al., 2014b) or a biological agent (e.g., virus or bacterial species) in known quantity (e.g., titer or cell count) could be added into a sample prior to sample processing (i.e., cell lysis) and shotgun sequencing, so that the absolute quantity of metagenomics reads recovered from a sample can be quantified by benchmarking to the spiked-in internal control. These methods have been used to quantify gene and transcript abundance in microbial communities in the Amazon river plume (Satinsky et al., 2014a,b), bathypelagic marine bacterioplankton communities after Deep Horizon Oil Spill (Rivers et al., 2013) and costal water in the USA (Gifford et al., 2011).

The studies discussed above demonstrate the possibility of using NGS platforms for both quantification of the relative abundance of microbial genes or taxonomic groups in a sample or absolute quantification through the use of internal standards. Nevertheless, potential biases and uncertainties introduced by use of nucleic-acid based methods and NGS (especially shotgun amplicon sequencing) require in-depth consideration during data analyses and interpretation.

## Application of NGS to Analysis of Water Quality

To date, integrated multi-omics approaches encompassing genomics, metagenomics, metatranscriptomics and MPS of targeted genes (e.g., SSU rRNA genes) have been used to unravel the functions of microbial communities in a variety of freshwater (reviewed by Fondi and Liò, 2015; Franzosa et al., 2015) and marine environments (reviewed by Bourlat et al., 2013). For microbial water quality assessment, these multi-omics analyses have been used in combinations to investigate the microbial compositions and their ecological functions (e.g., dissemination of antibiotic genes and pollutant degradation) in the urban water cycle, such as lakes and rivers, engineered waste tailings ponds, sewage, water distribution systems and finished waters, amongst others (**Table 1**). These studies yield insights into the activities mediated by microbial communities in aquatic systems and potential biological risk factors in the form of specific microbial populations and their genes for virulence, toxin biosynthesis, or antibiotic resistance (AR), which can be important in microbial water quality management.

**Table 1:** Selected publications using NGS for microbial water research.

Reference	Samples characterized	Objectives/core findings	Types of NGS survey	NGS platform
McLellan et al., 2010	Sewage influent communities in two waste water treatment plants (WWTPs) in Milwaukee (Wisconsin)	Examined eight sewer samples; identified microbial species associated with human-fecal and sewer materials; identified rainfall and storm water as factors modulating species composition in sewer treatment facilities	16S rRNA gene (V6) amplicon survey	Roche 454
Vandewalle et al., 2012	Sewage influent communities in two WWTPs in Milwaukee (Wisconsin)	Examined 38 sewer samples; identified major bacterial taxa (*Acinetobacter, Aeromonas, Trichococcus*) associated with sewage infrastructure, investigated the role of chemical variables have on community dynamics	16S rRNA gene (V6) amplicon survey	Roche 454
McLellan et al., 2013	Sewage influent communities in WWTPs in 12 cities geographically distributed across USA	Examined microbial communities in 12 WWTPs located in different cities in the USA. Compared distribution of *Clostridiales* within 100+ published samples originating from other US WWTPs with that in animal fecal material; identified host-specificity of *Blautia* spp. (*Lachnospiraceae*) in human, chicken, and cattle	16S rRNA gene (V6) amplicon survey (oligotyping)	Roche 454
Shanks et al., 2013	Sewage influent communities in WWTPs in 13 cities geographically distributed across US	Examined microbial communities in 13 WWTPs; identified and classified microbiomes detected in sewers into three groups: fecal, sewage, and transient microbiomes	16S rRNA gene (V6) amplicon survey	Roche 454
Newton et al., 2015	Sewage influents communities in 78 WWTPs geographically distributed across USA	Examined microbial communities in WWTPs in comparison to that in human stool samples; established evidence of core microbiomes shared between humans and treatments plants, and provided statistical evidence linking enrichment of specific microbial families to obesity rates in some US cities	16S rRNA gene (V4–V5) amplicon	Illumina MiSeq (paired-end)
Ye and Zhang, 2011	Wastewater treatment plant	Investigated the presence and distribution of pathogenic bacteria in 14 WWTPs in Canada, USA, Singapore, and China	16S rRNA (V4) gene survey	Roche 454
Cai and Zhang, 2013	Influent and eﬄuent of wastewater treatment plants in Hong Kong	Detected and tracked human bacterial pathogens in two Hong Kong WWTPs (influent, activated sludge, and eﬄuent)	Metagenomics	Illumina HiSeq
Ibekwe et al., 2013	Mixed urban environment	Analyzed the diversity and distribution of pathogenic bacteria (Genus level) in Santa Ana River watershed, California	16S rRNA (V1–V2) gene survey	Roche 454
Lu et al., 2015	Sewage treatment system	Characterized community composition and distribution of bacterial pathogens in advanced sewage treatment systems	16s rRNA gene (V3–V4) survey; metagenomics	Roche 454; Illumina HiSeq
Nshimyimana et al., 2015	Tropical mixed urban environment	Determined the bacterial community composition (BCC) in 18 sites in Kranji Reservoir and Catchment in Singapore (*n* = 36); evaluated whether the BCC and distribution of sewage-associated taxa or pathogen-like bacteria varied as a function of site, land use, and water quality	16S rRNA (V3–V4) gene survey	Illumina MiSeq (paired-end)
Gomez-Alvarez et al., 2012	Water distribution simulator (received treatment either with free-chlorine or chloramine)	Compared the effect of two different water treatment regimens on microbial composition and function; identified genetic determinants of potential pathogens in treated water	Metagenomics	Roche 454
Bai et al., 2013	Two microbial communities in a quartz-sand packed filtration unit (different depths) used in underground water filtration in a water treatment plant in China	Metagenomic analyses of bacterial communities in groundwater filtration system. Detected genes encoding for aerobic degradation of aromatic compounds, nitrification and denitrification	Metagenomics	Illumina HiSeq
Huang et al., 2014	Water samples (*n* = 3) collected from a water treatment plant in Beihekou, China following chlorination, filtration (sand packed column) and drinking water	Observed reduced microbial diversity in water following chlorination, though diversity increased in end-point tap water after passage through distribution pipelines. Genomic determinants for virulence factors were detected in the metagenomes	16S rRNA gene (V3–V4) amplicon survey, metagenomics	Roche 454; Illumina HiSeq
Chao et al., 2013	Raw water and chlorinated water (*n* = 1 each) collected from a drinking water treatment plant located in Pearl River Delta area, China	Identified reduced microbial diversity and elevation of genes encoding stress responses (e.g., oxidative stress) in chlorinated water versus raw water	Metagenomics	Illumina HiSeq
Shi et al., 2013	Filtered water and chlorinated water collected from a water treatment plant in Beihekou, China plus a tap water sampled in Nanjing, China (*n* = 1 each)	Identified an elevated representation of antibiotic resistance genes in chlorine treated water versus filtered water and tap water	Metagenomics; PCR (cloning and sequencing) and qPCR targeting antibiotic resistance genes	Illumina HiSeq
Chen et al., 2013	Metagenomic Profiles of antibiotic resistance genes (ARGs) in the Human Impacted Pearl River Estuary, China and sediments from the South China Sea	Comparative metagenomics of antibiotic resistance genes suggest enrichment of mobile genetic elements and high resistance potential to sulfonamides, fluoroquinolones, aminoglycosides, and beta-lactams in a human impacted estuary relative to deep ocean sediments	Metagenomics	Illumina HiSeq
Li et al., 2015	Metagenomic and network analysis of environmental antibiotic resistance genes in water, soil, sediments, wastewater, sludge, human fecal samples (*n* = 50)	Analysis of antibiotic resistance genes showed that heavily anthropogenic-impacted environments had a higher abundance (i.e., three orders of magnitudes) of ARGs associated with resistance to aminoglycosidase, bacitracin, beta-lactam, chloramphenicol, MLSB, quinolone, sulphonamide, and tetracycline	Metagenomics	Illumina HiSeq
Steffen et al., 2012	Comparative analyses of three metagenomes obtained for cyanobacterial blooms in Lake Erie, Lake Taihu, and Grand Lake St. Mary (*n* = 3)	Characterized the microbial composition of cyanobacterial blooms and their potential functions in three freshwater lakes; Identified genomic islands absent in freshwater metagenomes by mapping metagenomics reads to a reference genome of *Microcystis aeruginosa*	Metagenomics	Roche 454
Penn et al., 2014	*Microcystis* bloom in a tropical water reservoir over a 1-day/night cycle (*n* = 6)	Examined effect of diel cycle on gene expression of a *Microcystis* bloom; identified genes differentially expressed during day versus night, and genes encoding secondary metabolite biosynthesis	Metatranscriptomics	Illumina HiSeq GAII
Steffen et al., 2015	Water samples collected during daylight within 24 h from three sites in the western basin of Lake Erie	Examined community gene expression through metatranscriptomics analyses of waters containing elevated cyanobacterial biomass. Surveyed sites showed variations in microbial community structure but otherwise highly similar functional profiles. Transcription of genes implicated in nutrient cycles (i.e., nitrogen and phosphorous metabolism and urea degradation) varied according to sites and was linked to differing nutrient levels	Metatranscriptomics	Illumina HiSeq
Li et al., 2011	Cyanobacterial bloom in Lake Taihu (*n* = 1)	Identified non-cyanobacterial bacterial species present in a cyanobacterial bloom by excluding metagenomics contigs mapped to a reference genome of *Microcystis aeruginosa* isolated from the same system. Non- *Microcystis* reads were subsequently assigned to their putative functions to infer community functions	Metagenomics	Roche 454
Mou et al., 2013	Microcosms established using lake water collected from Western Basin of Lake Erie to study microcystin degradation by lake microbial community	Comparative metagenomics of two microcosms (amended with microcystin versus unamended control) identified microbial species and genes preferentially enriched during biodegradation of microcystin	Metagenomics	Roche 454
Hug et al., 2012	Three dechlorinating cultures established from underground water capable of degrading chlorinated ethene	Comparative metagenomics was used to identify microbial species that were likely involved in providing essential nutrients to Dehalococcoidetes during dechlorination of chlorinated ethene	Metagenomics	Sanger, Roche 454, Sanger + Roche 454
Luo et al., 2014	Benzene degrading microcosm prepared using soil and underground water from a decommissioned gasoline station	Comparative metatranscriptomic analyses identified putative genes involved in benzene degradation	Metatranscriptomics	Roche 454, Illumina HiSeq GAII
Tan et al., 2015	Two hydrocarbon enrichment cultures established from an oil sands tailing pond and another from a gas-condensate contaminated aquifer	Comparative analyses of hydrocarbon-degrading cultures identified microbial species and putative genes required in anaerobic degradation of hydrocarbons (i.e., aliphatic alkanes, monoaromatics, and polyaromatics)	16S rRNA gene (V6–V8) amplicon survey, metagenomics	Roche 454 and Illumina HiSeq
Laban et al., 2015	Toluene-degrading enrichment culture established from oil sands tailings pond	Identified key toluene degrader using toluene-stable isotope probing	Stable-isotope probing followed by metagenomic sequencing of heavy fraction	Illumina-MiSeq
Hemme et al., 2010	Heavy-metal contaminated ground water	Observed that a microbial community with limited diversity maintained a repertoire of genes and functions relevant to resistance to organic and inorganic pollutants	Metagenomics	Sanger
Smith et al., 2012	Underground microbial communities from a confined aquifer compared to an unconfined aquifer (with regular recharge events)	Comparative analyses identified metabolic functions differentially enriched in a confined versus an unconfined aquifer (with elevated iron, sulfur, total organic carbon, salinity, and pH) that may be related to strategies required for adaptation and survival	Metagenomics	Roche 454

*WWTP, waste water treatment plant; ARG, antibiotic resistance genes.*

In this section we review recent studies that have leveraged NGS to improve upon the current understanding of factors mediating water quality in mainly freshwater environments. In particular, we will discuss ongoing work to (i) identify indicators of human sewage and human fecal contamination for water quality assessment (see Discovery of New Indicators for Human Sewage Contamination), (ii) examine the fate and transport of human pathogens in water and wastewater systems (see Relationship between Fecal Indicator Bacteria and Pathogen-Like Sequences and Microbial Safety of Drinking Water), (iii) understand the ecological drivers of harmful algal bloom persistence and toxin production/degradation (see Toxin Production and Degradation in Cyanobacterial Blooms), (iv) observe the spread and distribution of AR in freshwater environments (see Tracking Antibiotic Resistance through Metagenomics), and (v) investigate mechanisms of natural and stimulated biodegradation of harmful pollutants that threaten water quality (see Understanding Biodegradation of Pollutants that Threaten Water Quality). While NGS-based studies are providing unprecedented insights into the structure and function of waterborne microbial communities, an open challenge remains to convert trends observed in these studies into actionable data for water quality managers.

### Discovery of New Indicators for Human Sewage Contamination

Contamination of freshwater bodies intended for human consumption or recreational use by fecal materials continues to be a major public health concern in many countries (Figueras and Borrego, 2010). New approaches are being developed to establish evidence of fecal contamination in surface freshwaters through alternative DNA-based indicators. MPS of the SSU rRNA gene has been used to characterize the microbial composition in raw sewage entering wastewater treatment plants in several different countries. Bacterial taxa associated with sewage infrastructure could be differentiated from those present in human fecal materials and other environmental sources (McLellan et al., 2010; Unno et al., 2010; Shanks et al., 2013; Cai et al., 2014). Comparison to a database of microbial sequences from feces in the Human Microbiome Project^1^ suggested that only ∼10–15% of sewage microbiomes in these surveyed sites were of human-fecal origin (McLellan et al., 2010, 2013; Shanks et al., 2013; Newton et al., 2015). Microbiomes of human-fecal origin detected in all sewage treatment plants appeared to be largely congruent and were represented by Firmicutes (e.g., *Lachnospiracea, Ruminococcaceae*; McLellan et al., 2013; Shanks et al., 2013), Bacteroidetes (e.g., *Bacteroidaceae, Porphyromonadaceae, Prevotellaceae*; Newton et al., 2015) and to a lesser extent by other mostly anaerobic microbes, e.g., *Bifidobacteriaceae, Coriobacteriaceae* (McLellan et al., 2010; Shanks et al., 2013; Newton et al., 2015). Amplicon sequences (V4–V5) obtained from municipal sewage communities and human stool samples identified 27 human fecal oligotypes (predominantly *Bacteroidaceae, Prevotellaceae*, or *Lachnospiraceae*/*Ruminococcaceae*) that are commonly and abundantly present in most surveyed sewage treatment facilities across the USA (Newton et al., 2015). The relative abundance of sequences within the dominant families of *Bacteroidaceae, Prevotellaceae*, or *Lachnospiraceae*/*Ruminococcaceae* could be statistically correlated to the obesity rate of the surveyed cities; with higher obesity rates corresponding to higher representation of *Bacteroidaceae* in the sewage microbiomes. This finding is particularly interesting as high representation of *Bacteroidaceae* in the human gut microbiome has been previously linked to consumption of a diet high in fat (David et al., 2014).

In contrast, a major proportion of the microbiome associated with sewage appeared to be adapted to the sewage infrastructure (∼80%) and varied in diversity and abundance according to geographical location (i.e., latitude) and air temperature (Vandewalle et al., 2012; Shanks et al., 2013; Newton et al., 2015). The microbiomes associated with sewers were also predominantly unique in taxonomy compared to those associated with animal hosts, surface freshwaters and other environmental sources (McLellan et al., 2010, 2013; Vandewalle et al., 2012; Shanks et al., 2013), and had in general higher diversity and species richness compared to stool samples (Newton et al., 2015). Such microbial species found in either human feces or sewage, but either absent, or present in very low abundance, in surface freshwaters, are potentially good indicators for sewage contamination (Koskey et al., 2014; McLellan and Eren, 2014). For example, development of qPCR probes based on *Lachnospiraceae* sequences enriched in human sewage were used to evaluate sewage contamination in an urban freshwater harbor where occurrence of this new indicator group was highly correlated with a more established marker of human fecal contamination (i.e., HF183; Newton et al., 2011). Analysis of profiles of microbial SSU rRNA genes (V4/V6 regions) with a source estimation program employing Bayesian statistics (SourceTracker) allowed identification of human fecal and sewage signatures which correlated to the distribution of human-source markers Lachno2 and HF183 along the coastline of Lake Michigan (Newton et al., 2013). Use of bacterial taxonomic groups identified through NGS-based surveys as alternative indicators may be site-specific as several studies have shown that the microbial composition in different sewer systems can differ according to several instances: origin of the waste materials (Ye and Zhang, 2013), climatic variations due to latitudinal locations of treatment facilities (Shanks et al., 2013), or infiltration of rainwater and storm water inputs (McLellan et al., 2010).

Collectively, studies using MPS of the SSU rRNA gene have shown that wastewater treatment is, in general, capable of removing most microbial populations associated with human-feces (Ye and Zhang, 2011; Cai et al., 2014). Even so, a small proportion of potential pathogens may still be present in sewer eﬄuents (Ye and Zhang, 2011; Cai et al., 2014). In areas without widespread sewage treatment, direct contamination of waters with human waste is still a problem. The cited studies have shown that assessment of waterbodies using NGS can reveal impacts from sewage and fecal contamination. In addition, with advancement in high throughput sequencing technology and bioinformatics, surveys of the sewage microbiome may 1 day be used to assess and monitor the overall health status of human populations.

### Relationship between Fecal Indicator Bacteria and Pathogen-Like Sequences

While NGS has shown potential for tracking water impairment through discovery and detection of indicators of human fecal or sewage pollution, a more direct approach to evaluate waterborne biological risks is through evaluation of pathogen diversity and abundance. However, tracking individual waterborne pathogens is costly, methodologically challenging, and requires knowledge of which pathogens to target (Varela and Manaia, 2013). The use of NGS to screen for sequences with high identity to waterborne pathogens allows identification of potential risk agents and, depending on biases in sample preparation, may also provide semi-quantitative inference of their relative abundance in wastewater and other environments (McLellan et al., 2010; Cai and Zhang, 2013; Ibekwe et al., 2013; Cai et al., 2014; Lu et al., 2015). Identification of pathogen species was not possible in early studies that were based on analysis of the V6 region of the SSU rRNA gene due to short sequence length (∼60 bp) which failed to provide taxonomic resolution beyond the family or genus level. However, as sequence read lengths through NGS continues to increase, confident classification of NGS-generated sequences to the level of bacterial species has become attainable.

A recent study of bacterial pathogens in a wastewater treatment plant applied a combination of NGS and qPCR of genetic markers to track the occurrence of bacterial pathogens through stages of wastewater treatment process (Lu et al., 2015). This analysis revealed that raw sewage was enriched in potential pathogens most closely related to *Arcobacter butzleri, Aeromonas hydrophila*, and *Klebsiella pneumonia.* The *Arcobacter* genus represented over 43.5–97.37% of all pathogen-like sequences in the treatment plant (sewage influent, primary eﬄuent, activated sludge, secondary eﬄuent and final sand filter eﬄuent) while the eﬄuent contained only *Arcobacter butzleri* with other non-detected species presumed to have been removed mainly by biological processes during treatment. Quantification of genetic markers with qPCR confirmed the trends in the distribution of pathogenic species identified by sequencing in both raw and treated sewage (Lu et al., 2015). In a previous study of sewage treatment plants across nations (Canada, USA, China, and Singapore), 454 pyrosequencing revealed that the most abundant sequences related to pathogenic bacteria in raw sewage corresponded to the genera *Aeromonas* and *Clostridium* with species *Aeromonas veronii, Aeromonas hydrophila, Clostridium perfringens*, and *Corynebacterium diphtheria* (Ye and Zhang, 2011).

Several studies have employed NGS to characterize the distribution of potential pathogens in natural and human-impacted areas. Nine sites sampled in urban and agricultural watersheds along the Santa Ana River, CA, USA were screened for potential bacterial pathogens by pyrosequencing of the V1 and V2 hypervariable regions of the SSU rRNA gene (Ibekwe et al., 2013). Sequences related to human pathogens comprised a greater percent of the sampled microbial community in urban runoff and agricultural waters than in waters collected from sites with little to no human activity. Similarly, a study of an urban tropical area in Singapore used Illumina MiSeq (V3 and V4 hypervariable regions of the SSU rRNA gene) to evaluate the variation of bacterial communities as a function of land use and water quality (Nshimyimana et al., 2015). The relative abundance of pathogen-like sequences identified to genera and species varied with land use and with the abundance of FIB, where moderate positive correlation to measured levels of *E. coli* suggested higher incidence of potential pathogens in sites with higher measured levels of FIB.

Despite the capability for simultaneous detection of multiple human bacterial pathogens in environmental waters, NGS has not yet been integrated into a framework of QMRA, in part because, as discussed in Section 1, (1) amplification-based biodiversity screens are qualitative, although studies of mock communities indicate that these may provide tentative indications of relative abundance, (2) identification of pathogens to species-level has only recently been possible with longer reads afforded by NGS, and (3) species-level identification may not be sufficient to assess risk from pathogens with strain-specific virulence.

Recent studies have attempted to monitor human sewage to track outbreaks of disease-causing viruses. While published studies have not yet integrated NGS, such methods could complement conventional approaches such as PCR, qPCR, and Sanger sequencing for the identification and quantification of pathogens. This method combination was recently employed for analysis of eight viral strains in sewage in Gothenburg, Sweden and led to an early warning of a potential outbreak of Hepatitis A and norovirus (Hellmér et al., 2014) which were also detected in an ongoing outbreak in Scandinavia (Hellmér et al., 2014). Clinical research and diagnostic studies in Africa also combined three methods (Sanger sequencing, PCR, and qPCR) to understand the spread and distribution of the polio virus and to discern wild-type from vaccine-derived strains (Gumede et al., 2013). Future studies have the potential to apply NGS to screen for and identify pathogens that could subsequently be monitored by targeted diagnostic methods such as PCR and qPCR.

Reviewed studies demonstrate that NGS and related sequence-based methods are being used to track human bacterial pathogens and/or waterborne viral strains. Monitoring the distribution of viral strains in the environment shows promise to improve vaccination campaigns and provide early warnings of disease outbreaks in a given community. NGS profiling of bacterial pathogens in the natural and built environment provides general insights into how the distribution and relative representation of potential human pathogens varies with environmental conditions, anthropogenic impacts, and the implementation of water treatment technologies. Such information may be leveraged to guide engineers and watershed managers in selection of best practices to reduce human exposure to potential pathogens.

### Microbial Safety of Drinking Water

**FIGURE 1:**
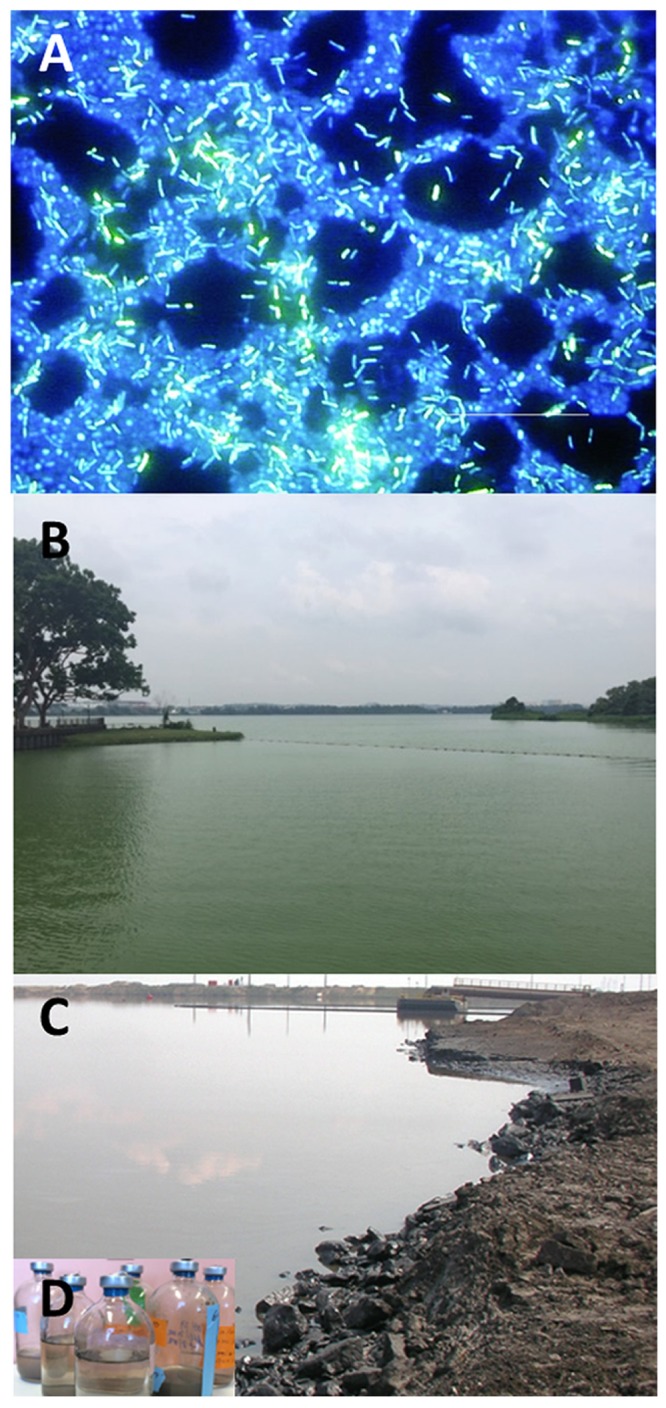
**Environments where studies of water quality have been advanced by NGS. (A)** Fluorescence microscopy showing biofilm grown on a stainless steel surface in a laboratory potable water biofilm reactor for 14 days (image from Donlan, 2002). Drinking water is thought to contain a mixture of both planktonic and dissociated biofilm bacteria. Using NGS methods, a repertoire of microbial species including potential pathogens have been detected in water distribution systems, raising the concern of the effectiveness of water treatment on microbial water safety. **(B)** A water reservoir in Singapore impacted by a cyanobacterial bloom characterized by Penn et al. (2014). With global warming, cHAB is predicted to occur more regularly with likely severe consequences (e.g., toxin loading) that can diminish water quality. **(C)** An oil sands tailings pond in Alberta, Canada characterized by Tan et al. (2015). Inset **(D)** shows microcosms established to study degradation of unrecovered hydrocarbons deposited in oil sands tailings pond. NGS has been used to characterize the microbial communities in oil sands tailings ponds (http://www.hydrocarbonmetagenomics.com/) in order to better understand mechanisms of pollutant degradation.

Next-generation sequencing has been used to survey the microbial composition in drinking water distribution systems including source waters (Chao et al., 2013), end-point taps (Chao et al., 2013; Shi et al., 2013; Huang et al., 2014), and various stages of the drinking water treatment and distribution process (Gomez-Alvarez et al., 2012; Chao et al., 2013; Shi et al., 2013; Huang et al., 2014), as well as biofilm matrices associated with distribution pipelines (**Figure 1A**; Hong et al., 2010). While most microorganisms in treated drinking water are harmless, outbreaks of diseases linked to pathogens in drinking water may be due to compromised water treatment and contaminated source waters (MacKenzie et al., 1994; Howe et al., 2002). Pathogens that have evaded treatment and disinfection processes may persist in water distribution pipelines as biofilms, leading to dissemination to end users through the process of sloughing (Figueras and Borrego, 2010). A recent study noted an increase in microbial diversity in end-point drinking water relative to source water which was linked to dispersal of biofilm-associated microbes during passage through the water distribution pipeline (Huang et al., 2014). Consumption of untreated well-water poses an additional risk, where waterborne pathogens such as *Legionella* and *Campylobacter* are among some of the main well-waterborne disease agents in the USA (CDC, 2013). Other causative agents of waterborne diseases in both developed and developing countries include parasites such as *Cryptosporidium parvum, Toxoplasma gondii, Cyclospora cayetanesis, Giardia lamblia* and viruses such as norovirus (Ashbolt, 2015). Metagenomic surveys of water within distribution systems and end-point drinking waters have detected DNA indicative of several of these potential opportunistic pathogens (e.g., *Legionella, Mycobacterium, Pseudomonas*, and *Leptospira*) and virulence factors (e.g., AR and pathogenicity islands, Gomez-Alvarez et al., 2012; Shi et al., 2013; Huang et al., 2014). Several recent studies have used NGS to shed light on the fate of microbial populations, including pathogens, during various stages of the water treatment process.

Microbial communities in drinking water systems tend to be present at low abundance (e.g., <10^5^ cells per ml, personal observation) hence studies of microbial communities in drinking water require collection of microbial biomass from large amounts of water, e.g., 100–2000 L (Gomez-Alvarez et al., 2012; Chao et al., 2013; Shi et al., 2013; Huang et al., 2014) where DNA extraction yields may still be too low for direct sequencing, requiring amplification (of genomes or amplicons) prior to sequencing (Gomez-Alvarez et al., 2012). Studies of water distribution systems reveal microbial communities dominated by the phyla Proteobacteria (i.e., Alpha-, Beta-, and Gammaproteobacteria), Firmicutes, Nitrospirae, and Actinobacteria (Gomez-Alvarez et al., 2012; Chao et al., 2013; Huang et al., 2014). Application of disinfectants (e.g., free chlorine or monochloramine) appear to have selective effects on the microbial assemblages in water distribution pipelines and end-point drinking water (Gomez-Alvarez et al., 2012; Chao et al., 2013; Huang et al., 2014). In water treated with monochloramine collected from a water distribution simulator in the USA, the microbial composition based on taxonomic assignment of 454 reads, was dominated by Actinobacteria (28%; e.g., *Mycobacterium*), Betaproteobacteria (25%; e.g., *Acidovorax*), and Alphaproteobacteria (23%), whereas free-chlorine treated water had a lower proportion of Actinobacteria (6%), and was dominated largely by Alphaproteobacteria (35%; e.g., *Caulobacter, Rhodopseudomonas, Bradyrhizobium*) and Cyanobacteria (*Synechococcu*s; Gomez-Alvarez et al., 2012). Chlorination appeared to selectively deplete Gamma- and Betaproteobacteria, resulting in higher relative abundance of Alphaproteobacteria in drinking water (Chao et al., 2013; Huang et al., 2014). Relative to untreated water, treatment with chlorine was found to reduce waterborne microbial diversity (Huang et al., 2014). Importantly, chlorination was associated with the removal of most potential pathogens from the detected diversity (Huang et al., 2014) although genomic signatures of some potential pathogens (e.g., *Pseudomonas aeruginosa* and *Leptospira interrogans*) persisted in the tap water (Huang et al., 2014). Protective functions for detoxification and modulation of oxidative stress (Chao et al., 2013), as well as mobile genetic elements (MGEs; Shi et al., 2013) were also found to be more highly represented in metagenomes obtained from water following disinfection (Shi et al., 2013; Huang et al., 2014).

### Toxin Production and Degradation in Cyanobacterial Blooms

Cyanobacterial harmful algal blooms (cHABs) are destructive to aquatic ecosystems, deteriorating surface water quality and rendering it unsafe for use by humans and livestock. cHABs are associated with overgrowth of cyanobacterial species many of which are capable of producing cyanotoxins, e.g., microcystin, cylindrospermopsin, anatoxin (Ferrão-Filho and Kozlowsky-Suzuki, 2011) and off-flavor/odorous compounds, e.g., geosmin or 2-methylisoborneol (Li et al., 2012). Rising global temperature as a result of climate change is expected to promote the frequency and severity of cHAB events in the future (Paerl and Huisman, 2009). NGS technologies are being applied to the systematic understanding of the ecology and control of cHAB using methods in metagenomics, comparative genomics, and metatranscriptomics (Li et al., 2011; Steffen et al., 2012, 2015; Penn et al., 2014).

A cyanobacterial bloom consists of both primary producers and heterotrophs including consumers and grazers, resulting in nutrient cycling and recycling. It is likely that different populations of microbial heterotrophs may influence the dynamics of a cHAB and may benefit from nutrients and metabolites released during a cHAB event (Edwards and Lawton, 2009; Mou et al., 2013), or consumption of dead cyanobacterial biomass after a bloom die-off (Xing et al., 2011). The interplay of this complex ecosystem shapes the bloom community structure and may control the adverse effects of the bloom (e.g., bloom persistence and the balance between production and biodegradation of toxins and off-flavor/odor compounds). Though this interplay has long been hypothesized (Christoffersen et al., 1990), detailed insights from community gene expression can link specific populations, or groups of populations, to specific activities via metagenomic and metatranscriptomic approaches. In contrast to oligotrophic lakes where phototrophic Proteobacteria and Actinobacteria appear to be the most active light harvesters (Yurkov and Beatty, 1998; Sharma et al., 2008), in a cHAB, which generally manifests during eutrophic conditions, solar energy is captured primarily by algae and cyanobacteria. Metagenomic studies conducted during bloom events in Lake Taihu (China), Lake Erie (N. America), and Grand Lake St. Marys (GLSM; OH, USA) revealed a high proportion of cyanobacterial sequences (25–88%) including common bloom members *Chroococcales* (e.g., *Cyanothece, Synechococcus, Crocosphera;* ca. 40–45%), *Nostocales* (ca. 10%), and *Oscillatoriales* (e.g., *Lyngbya, Trichodesmium*-like, *Arthrospira*, etc; ca. 17–38%; Steffen et al., 2012). Metatranscriptomics studies of other freshwater systems suggest that the most active cyanobacterial taxa were also from the same cyanobacterial orders, dominated by *Microcystis* in an eutrophic Singaporean reservoir (Penn et al., 2014; **Figure 1B**) and in Lake Erie with co-dominant taxa including *Synechococcales* and *Gloeobacterales* (Steffen et al., 2015). Several recent -omics studies showed that the non-cyanobacterial members of cHAB communities were dominated by Proteobacteria (>90% of non-cyanobacterial sequences) with a smaller portion of Bacteroidetes (Li et al., 2011; Steffen et al., 2012; Penn et al., 2014). Eukaryotic algal species (e.g., *Streptophyta, Euglenids, Chlorophyta, Bacillariophyta*) were also observed within blooms, and may co-dominate, as revealed using MPS of the plastid 23S rRNA gene, specific for cyanobacterial and eukaryotic algal species (Steven et al., 2012). In addition, viruses/phage, protozoa, and fungi are present and are increasingly recognized as key players in bloom persistence and decay (Gerphagnon et al., 2013; Xia et al., 2013; Ger et al., 2014).

Studies based on NGS have provided unprecedented insights into the evolution of cyanobacterial genomes, pointing to the role of nutrient-enrichment as a factor that can accelerate genome evolutionary rates and potential niche expansion of cyanobacterial species. Comparative genomic analyses reveal signatures of genome rearrangement through both homologous and non-homologous recombination (Frangeul et al., 2008; Humbert et al., 2013; Yang et al., 2015) with such variation reflected in natural bloom populations. Comparison of the genome of *Microcystis aeruginosa* strain NIES 843 to metagenomes from Lake Erie, Lake Taihu, and GLSM identified metagenomic islands (Mi’s) within the NIES 843 genome (defined as regions of ≥10 kb with low coverage in the metagenomes) that were not observed in these three sites and which contained transposase and MGEs (Steffen et al., 2012). Variation in toxin biosynthesis gene content among closely related cyanobacterial strains is well-documented (Mikalsen et al., 2003; Tanabe et al., 2004; Christiansen et al., 2008) and is linked to genomic dynamism mediated by homologous and non-homologous recombination. Transposase expression in strain NIES 843 has been shown to be upregulated by nutrient-enrichment, particularly in the presence of organic nitrogen (urea; Steffen et al., 2014). Thus, conditions of eutrophication may promote genome rearrangement within this group, contributing to the mosaic patterns in gene order, MGE content and the distribution of toxin genes observed in these strains. The dynamic genomes of cHAB strains may influence their fitness, promoting local adaptation and contributing to the widespread distribution of closely related strains (Mikalsen et al., 2003; Yoshida et al., 2008; Acinas et al., 2009).

It is of interest to water quality managers to know which waterborne microbial populations are responsible for the production and biodegradation of toxins and off-flavor/odorous compounds. Transcripts for genes in the biosynthesis pathways for multiple cyanotoxins (i.e., microcystin, aeruginosin, and cyanopeptolin), and structurally related compounds including a newly identified secondary metabolite gene cluster were detected throughout a 24 h sampling campaign in Singapore, suggesting that toxin gene expression was continuous in this system, despite dissolved toxin levels measured near the limit of detection (Penn et al., 2014). Biosynthesis and release of toxins may be balanced by their biodegradation. Biodegradation of microcystin by natural microbial communities from Lake Erie was studied through metagenomic analysis of mesocosms (Mou et al., 2013). The authors found *Methylophilales* and *Burkholderiales* were significantly enriched in microcystin-amended microcosm, while the gene encoding for the only known mechanism of microcystin biodegradation (mlr) was not, prompting the authors to speculate that new, undiscovered pathways for microcystin biodegradation were being utilized. As additional omics-enabled studies with a focus on water quality emerge, it is likely that new pathways for the production and biodegradation of such compounds will be discovered, paving the way for better strategies to control their expression and improve source water quality.

### Tracking Antibiotic Resistance through Metagenomics

Antibiotic resistance in pathogenic bacteria is a growing public health threat (Berendonk et al., 2015). Antibiotic resistance determinants (ARDs) including antibiotic resistance genes (ARGs), and MGEs that catalyze the transfer of such genes, occur widely in environmental bacteria. ARD appear to be enriched in microbial communities including sediment microbiota near wastewater treatment plant eﬄuent outfalls (Czekalski et al., 2014; Port et al., 2014), sediments of human-impacted estuaries (Chen et al., 2013) and in a variety of sample types (e.g., soil, water, sediments, human and animal fecal samples, sludge, wastewater) derived from heavily anthropogenic impacted environments (Li et al., 2015). Although environmental surveillance of AR is not currently part of water quality monitoring frameworks, there is an urgent need to better control the spread and evolution of AR and a better understanding of the occurrence and characteristics of environmental reservoirs of ARD will advance that goal (Bush et al., 2011).

To gain a better understanding of the types of ARGs in environmental bacteria and their co-localization with MGEs (e.g., transposons, plasmids, and integrons) complementary approaches of sequence-based metagenomics and functional metagenomics has overcome the impediments of earlier culturing and molecular techniques. With sequence-based metagenomics, total DNA from an environment is directly extracted and randomly sequenced. The sequenced reads are then interrogated against a reference database containing known ARG sequences to predict the resistance potential originating from a metagenome. Functional metagenomics involves cloning randomly sheared DNA fragments into an expression vector and transforming them into a host (e.g., *E. coli*) and selecting transformants which exhibit resistance to the selected antibiotic (Allen et al., 2009; Sommer et al., 2009). The advantages of this dual approach include (i) the ability to identify highly divergent genes from known ARGs; (ii) direct evidence of resistance phenotypes associated with expressed genes and (iii) no reference gene sequences required for gene identification (Pehrsson et al., 2013). Combining sequence based metagenomics and functional metagenomics has led to the discovery of novel ARGs in various microbial communities including soil (Riesenfeld et al., 2004; Allen et al., 2009; Torres-Cortés et al., 2011), freshwater lakes (Bengtsson-Palme et al., 2014), human gut microbiomes (Sommer et al., 2009; Hu et al., 2013), oral microbiome (Diaz-Torres et al., 2006), animal gut microbiomes (Kazimierczak et al., 2009) and activated sludge (Mori et al., 2008; Parsley et al., 2010).

The growing sophistication, accuracy and speed in downstream processing pipelines of NGS datasets provides a significant step forward in facilitating large-scale environmental studies to assess the emerging threat imposed by AR. With the inundation of metagenomic sequence data over the past decade from variety of environmental microbiomes (e.g., IMG, MG-RAST, Sequence Read Archive), metagenomic analysis tools have been developed to mine larger and more expansive reference sequence databases for ARG signatures. These tools include the Comprehensive Antibiotic Resistance Database (CARD^2^, McArthur et al., 2013), the Antibiotic Resistance Database (Liu and Pop, 2009), the beta-lactamase database (BLAD, Danishuddin et al., 2013), and ResFinder^3^. To determine the relationship between environmental and human-associated resistomes (i.e., antibiotic resistance genes and bacteria), Gibson et al. (2015) developed Resfams, a curated protein family database and associated profile hidden Markov models (HMMs), organized by ontology specifically applied to AR functions, with a subset of these AR proteins functionally verified using protein assays. This method circumvents the common approach of assigning AR functions using pairwise sequence alignment to AR databases, instead, HMM and consensus models were used for AR functional assignment, which significantly increases prediction sensitivity and specificity.

Several recent studies have employed NGS and bioinformatic tools, such as those described above, to characterize the AR profiles of microbial communities in aquatic environments. A network analysis was conducted to investigate the broad-spectrum profiles of ARGs and their co-occurrence patterns in 50 samples spanning water, soil, sediments, wastewater, sludge, and human and fecal samples. This study concluded that the abundant ARGs were associated with antibiotics commonly administered in human or veterinary medicine (i.e., aminoglycoside, bacitracin, beta-lactam, chloramphenicol, macrolide-lincosamide-streptogramin, quinolone, sulphonamide, and tetracycline) and abundances of these ARGs were up to three magnitudes higher in the most heavily anthropogenic-impacted environments (Li et al., 2015). Similarly, metagenomic profiles of ARGs in sediments of a heavily human impacted estuary [Pearl River Estuary (PRE) in China] revealed a higher diversity of both genotypes and resistance genes for sulphonamides, fluoroquinolones, and aminoglycosides relative to a pristine deep ocean bed in the South China Sea (SCS; Chen et al., 2013). In addition, this study showed parallel trends between the distribution of ARGs and MGEs, where MGE’s may function as vectors for dissemination of ARGs in the aquatic environment.

Antibiotic resistance is a public health threat of heightened concern and the central theme of current global monitoring efforts is limited to tracking antibiotic consumption and antibiotic resistant bacteria (ARBs) isolated from clinical and public health laboratories (Grundmann et al., 2011). While clinical settings may be the source of highest antibiotic and ARB loads, the natural environment has recently drawn attention as a reservoir of transferable ARGs potentially implicating environments highly contaminated with ARGs as a human health risk (Ashbolt et al., 2013). ARGs in particular are increasingly viewed as emerging pollutants and emphasis of their occurrence and distribution in natural environments are being considered in development of antibiotic surveillance frameworks (Codex Alimentarius Commission, 2011; Berendonk et al., 2015). Incorporating metagenomics into frameworks for monitoring the threat of AR in aquatic environments provides a novel approach for environmental health monitoring and pushes boundaries on improving current risk assessment models. Recently, a metagenomic-based approach was used to develop a multivariate index to quantify the AR potential in published metagenomes (Port et al., 2014). This index was based on the abundance of ARGs, MGEs, pathogenic potential and metal resistance genes (implicated in co-selection of ARGs). Congruent with studies examining the distribution of ARG, the multivariate index was shown to differentiate aquatic environments based on human-impact. This study exemplifies the utility of NGS to advance characterization of water quality within the context of AR, which will ultimately assist in risk evaluation and mitigation by public health authorities.

### Understanding Biodegradation of Pollutants that Threaten Water Quality

Chemical pollution is one of the major causes of diminished water quality (Schwarzenbach et al., 2010). Strategies for pollutant removal through stimulation of indigenous microbial community for bioremediation can be effective to remove, or immobilize, toxic compounds (Major et al., 2002; Gieg et al., 2014; Koenig et al., 2014). The presence of pollutants can exert selective pressures that enrich for microbial populations that are capable of coping with associated stresses and/or are able to utilize chemical contaminants as a source for carbon, nutrients, or as an electron acceptor for respiration (Hemme et al., 2010; Smith et al., 2012; An et al., 2013a,b; Rivers et al., 2013; Tan et al., 2015). Recent work in the area of marine metagenomics has demonstrated that the enrichment of microbial genes in distinct environments reflects the key biogeochemical processes in these systems (Coleman and Chisholm, 2010; Ulloa et al., 2012; Kelly et al., 2013). Similarly, microbial communities and gene expression profiles in polluted waters reveal pathways for transformation of contaminants, thus serving as an indicator of *in situ* biodegradation processes and highlighting metabolic pathways to target and possibly stimulate for accelerated pollutant clean up (Kimes et al., 2013; Engel and Gupta, 2014; Lamendella et al., 2014; Mason et al., 2014).

Analysis of microbial populations and genes enriched in polluted sites using “-omics” enabled approaches has revealed key microbial processes involved in contaminant tolerance and transformation. For example, in an underground water system heavily polluted by heavy metals, nitric acid and organic solvents, the microbial community surveyed through metagenomics showed limited diversity consisting mainly of Beta- and Gammaproteobacteria, with streamlined metabolic capabilities including the capacity to utilize heavy metal ions for lithotrophy (Hemme et al., 2010). A similar link between microbial gene content and contaminant transformation is observed in systems contaminated with hydrocarbons. In the highly anaerobic environments of oil sands tailings ponds constructed to store waste tailings from bitumen extraction, metagenomic data identified a repertoire of facultative and strict anaerobes that were capable of utilizing the pollutant hydrocarbons as a carbon source (**Figure 1C**; An et al., 2013a; Tan et al., 2015). Similarly, NGS surveys of open oceans and coastal shorelines in Gulf of Mexico after Deep Horizon Oil spill in 2010 showed that within the water column, a microbial community consisting of mainly marine Gammaproteobacteria (e.g., *Colwelliaceae, Oceanospirillaceae, Piscirickettsiaceae, Methylococcaceae*) dominated the oil plumes, and were the key players in the aerobic degradation of oil and gas (Mason et al., 2012; Rivers et al., 2013). MPS of SSU rRNA genes from microbial communities originating from beach areas before and post-oil impact indicated that the community shifted from a baseline of predominantly enteric-type consortia associated with human-impact to marine-associated taxa (e.g., *Oceanospirillales, Rhodospirillales*, and *Rhodobacterales*) linked to remediation efforts (e.g., sand washing), including populations with potential oil degradation capability (Engel and Gupta, 2014). Oil plumes have been shown to have an impact on microbial communities in marine sediments and other beach areas, with NGS detecting genes and transcripts indicative of pollutant (monoaromatics and alkanes) degradation in marine sediments (Kimes et al., 2013; Mason et al., 2014) and areas along the shorelines (Lamendella et al., 2014).

Several studies have used microcosms and enrichment cultures inoculated from contaminated sites (i.e., polluted groundwater or tailings ponds containing toxic waste) to establish evidence of pollutant degradability, followed by NGS studies incorporating gene expression (i.e., metatranscriptomics) or comparative metagenomics approaches (Hug et al., 2012; Waller et al., 2012) in order to identify (novel) key processes relevant to pollutant degradation (e.g., Abu Laban et al., 2010; Hug et al., 2012; Luo et al., 2014; **Figure 1D**). NGS in tandem with other functional analyses (e.g., proteomics and transcriptomics) have been employed to successfully identify novel genes and microbes involved in anaerobic activation and degradation of recalcitrant compounds, e.g., carboxylation of benzene (Abu Laban et al., 2010; Luo et al., 2014), dechlorination of chlorinated ethene (Hug et al., 2012; Waller et al., 2012) and fumarate addition activation of hydrocarbons (Tan et al., 2015). Comparative metagenomics and genome-centric metagenomics have been used to tease out the functional roles of different members in complex community in bioreactors (Albertsen et al., 2013; Haroon et al., 2013). Microbially mediated processes, such as dechlorination of chlorinated compounds often requires the presence of a mixed community to provide supporting roles such as production of essential nutrients, e.g., H_2_, corrinoid, and methionine that are needed by primary degraders (e.g., Dehalococcoidetes) in substrate mineralization. Hug et al. (2012) compared the metagenomes of three enrichment cultures involved in the dechlorination of chlorinated ethene, and identified genes required for the production of nutrients essential for Dehalococcoidetes in dechlorination. Assigning these gene to taxa using a homology-approach; revealed a mutualistic relationship between a repertoire of microorganisms (e.g., Firmicutes, methanogens, and Deltaproteobacteria), which is not easily achieved using conventional microbiology methods involving isolation. The functions of key members involved in the degradation of pollutants could also be inferred through targeted genome enrichment using strategies such as stable-isotope probing (SIP), in which labeled recalcitrant compounds can be used to enrich for key degraders, followed by (meta)genome sequencing and analysis allowing investigation of the genetic capabilities or metabolic modeling of uncultivated pollutant degraders (e.g., Saidi-Mehrabad et al., 2013).

## Future Technologies on the Horizon and Impact for Water Quality Assessment

Next-generation sequencing platforms for single molecule real-time sequencing technology (SMRT; e.g., Pacific Biosciences and Oxford Nanopore Technologies) are capable of producing sequences with read lengths exceeding 1000 bp, surpassing those generated using Illumina and Roche 454. In addition, real-time data acquisition make these technologies attractive targets for integration into sensors for monitoring environmental sequence data. To date, these technologies have not been widely adopted for surveys of highly diverse bacterial communities due to high rates of randomly distributed sequencing errors, which would lead to artificially inflated community diversity (Schloss et al., 2015). However, recent studies employing SMRT sequencing with improved sequencer hardware and chemistry have yielded improved accuracy and read lengths (i.e., >1400 bp) making community surveys with amplicon sequencing more feasible (Marshall et al., 2012; Mosher et al., 2014). To date, Pacific Bioscience (PacBio) sequencing has been used extensively for genome sequencing, where the high coverage and long sequence read length allows for generation of highly contiguous consensus sequences with low error rates, where microbial genomes can often be closed within a single run (Koren et al., 2013). PacBio sequencing is often used in parallel with other NGS platforms (e.g., Illumina, Roche 454, and SoLiD) to allow for scaffolding and phishing to produce finished/close genomes with high sequence quality (Koren et al., 2013). MinION, a newer SMRT based on Nanopore technology (Venkatesan and Bashir, 2011), has garnered a lot of interest due to the cost-effectiveness and pocket-size mobility. Hundreds of units were shipped to members of the MinION Access Program to test the device in a wide range of sequencing applications. Sequencing results produced by some of the laboratories showed that MinION could generate single read lengths of up to 5500 bp in a single run, though riddled with a high sequence error rate (∼30%; Ashton et al., 2014; Mikheyev and Tin, 2014; Madoui et al., 2015). Nanopore sequencing holds particular promise for online detection of waterborne nucleic acids due to its high potential portability and real-time data output, allowing future work to develop real-time sensing platforms for water quality monitoring. Indeed, as different NGS technologies advance toward enhanced sequence chemistry for improved sequence read length with higher throughput and reduced error rate, online real-time detection using NGS will breach the gaps for its use in real-time monitoring of genetic parameters for water quality.

The synergy between multiple NGS platforms and the increased accuracy and data processing speed of downstream bioinformatics, biostatistical and machine-learning pipelines continue to push the boundaries of genomic and metagenomic data analyses. Advancements in the field of multi-omics have enabled optimization of bioinformatics workflows (Kozich et al., 2013; Nelson et al., 2014); allowing acquisition of uncultivated microbial genomes from a complex metagenome (Huson et al., 2007; Dick et al., 2009; Hess et al., 2011; Albertsen et al., 2013; Haroon et al., 2013); deduction of microbial community composition based on single copy genes (Sunagawa et al., 2013); inference of metabolic interactions among microbial community members (Hanson et al., 2014); identification of phylogenetic markers and genes encoding important processes (Stark et al., 2010; Darling et al., 2014), and from a broader public health aspect, have augmented our knowledge of the population genomics and dissemination of virulent strains of waterborne pathogens such as *Vibrio cholerae* (Dutilh et al., 2014).

The influx of newly sequenced microbiomes associated with various natural and engineered water environments progressively expand sequence information available in public databases such as MG-RAST (Meyer et al., 2008) and IMG (Markowitz et al., 2014), providing a bigger data pool for mining and comparative metagenomic analyses. This is evident in ecological studies of freshwater microbiomes where taxonomic composition and functions of waterborne microbial communities and pathogens are dependent upon *a priori* databases. In addition, integrating physiological methods through use of microcosms, enrichment cultures and single cell genomics, coupled with NGS has allowed the discovery of new enzymes and genes in complex microbial communities that are important in water quality preservation (e.g., toxin degradation, nutrient cycling), which otherwise would be overlooked since many of these microbes have remained uncultivated.

Microbial water quality assessment through NGS-based molecular detection of FIB, pathogens, or virulence factors, as well as genes encoding biogeochemical processes such as pollutant biodegradation, have the potential to be translated into actionable data for water quality managers. However, crucial relationships between the occurrence, detection and quantification of nucleic acids in the environment through NGS-based approaches and potential impacts to human or environmental health must be established before profiles based on the distribution(s) of microbial genes are relied upon to replace currently used bioindicators. Current frameworks for water quality assessment heavily rely upon proxy measurements (e.g., quantification of culturable FIB) with a deep literature substantiating such approaches with epidemiological data. As the field of NGS-based water quality assessment matures, such further studies will be needed to establish whether proposed NGS-based indicators for pollution can improve upon the existing state of the art in water quality assessment.

## Conflict of Interest Statement

The authors declare that the research was conducted in the absence of any commercial or financial relationships that could be construed as a potential conflict of interest.

## Footnotes

1 http://hmpdacc.org/

2 http://arpcard.mcmaster.ca/

3 https://cge.cbs.dtu.dk//services/ResFinder/
